# Comparative Analysis on the Micropore and Microstructure Characteristics of Concrete under Insulated Formwork

**DOI:** 10.3390/ma14112862

**Published:** 2021-05-26

**Authors:** Myung-Kwan Lim, Kyung-Yong Nam

**Affiliations:** 1 Department of Architectural Engineering, Songwon University, Gwangju 61756, Korea; limmk79@naver.com; 2 UTOP E&A, 48 Hwasun-eup, Jeollanam-do, Hwasun 58118, Korea

**Keywords:** concrete performance, insulated gang form, mechanical properties, chemical admixture

## Abstract

During concrete construction in winter, the concrete performance is generally improved by adding a chemical admixture or providing protection using tents and hot-air blowers. However, long-term strength or safety accidents may occur due to the installation and removal of the tents. This study considered insulated gang forms to improve formwork methods. In this regard, the microstructure and micropore characteristics of concrete were investigated experimentally to examine the insulated gang form effect on the physical and mechanical properties of concrete. The micropore characteristics were investigated through scanning electron microscopy. The results confirm that applying insulated gangs improves workability and safety without adding chemical admixture. Moreover, the application of insulated gang forms reduces the use of tents and hot-air blowers. Therefore, insulated gangs provide excellent initial quality to the concrete.

## 1. Introduction

In modern high-rise building construction, reducing the period of frame construction is essential for ensuring project feasibility. Therefore, selecting an appropriate formwork method is crucial [1].

Formwork construction represents a considerable proportion of the building construction cost as this step accounts for 30–40% of the structure construction cost and 10% of the overall construction cost [2,3,4]. In addition, the formwork method plays a crucial role in reducing the time required to raise one floor [1]. Moreover, selecting an appropriate formwork method is paramount as it affects the total construction period and cost, as well as the subsequent processes, such as electricity and equipment [5]. Therefore, there is a continuous interest in studying novel methods regarding formwork construction.

As a formwork method, gang forms have been mainly used for high-rise offices and apartments in various countries [6]. For such high-rise modern buildings, reducing the construction period is considered important. In particular, in cold countries where the average daily temperature falls below 4 °C, year-round construction has become a necessity. Moreover, in countries with distinct seasonality, concrete quality control has also been considered important [7].

Formwork and concrete construction in winter have the problem of preventing early frost damage in the concrete and securing early strength [8]. If early frost damage occurs before the concrete hardens, the compressive strength of the concrete reduces. In this regard, the compressive-strength reduction can be due to various causes; however, concrete significantly affects the resistance to early frost damage if its compressive strength reaches a certain target before occurring early frost damage [9,10,11].

There are several countermeasures to prevent such early frost damage [8]: (1) after pouring concrete, the area is enclosed in a tent with the inside heated with an oil stove or by electric heating, (2) water or aggregate is heated, (3) the content of cement or Type Ⅲ-R Portland cement is increased, (4) protective insulation is used, or (5) chemical admixture is used in winter to improve strength development at early ages. However, these methods are not generally applied in all countries.

Method (1) is the most typically used method; however, environmental problems, and problems such as the death of workers from suffocation, frequently occur due to the use of hot-air blowers and heaters. In Method (2), the cost of heating water and aggregate increases for ready-mixed concrete manufacturers. For (3), the cost increases owing to the increase in the amount of cement and change in cement type. For (4), protective insulation is used only for a part of the structure, and the labor cost and construction period increase when used for the entire structure. Finally, several studies have been conducted on method (5). As representative cases, Cullu and Arslan studied the physical and mechanical properties of concrete that used antifreeze in cold weather [12]. Riza Polat studied the effects of elements to secure the strength of fresh concrete in cold weather [8]. Lee investigated the use of accelerators to develop the early strength of fresh concrete [13].

Various types of chemical admixtures have been used to prevent early frost damage in fresh concrete in winter [14]. However, most of these studies have not been implemented due to inconveniences such as mix design adjustment, reduction in long-term strength, and chlorides. Method (1), which can directly control the curing temperature, has been mainly used [15].

However, some studies have pointed out that method (1) presents problems such as high fuel cost, increased installation cost, and environmental pollution [16,17]. Thus, studies on formwork have been conducted to improve these limitations. Most of the previous studies mainly focused on preventing frost damage in fresh concrete. In addition, several studies were conducted to evaluate the durability of hardened concrete, including the microstructure and microporous properties of the hardened concrete [18,19,20].

In other studies, the effect of temperature on the composition of pore solution was investigated [21,22,23,24]. These studies presented data on temperature dependence, quantity and stability of hydration products, and pore solution composition.

The temperature change, due to the heat of hydration or external environment change, significantly affects the mechanical properties of concrete at an early age. Therefore, it is necessary to investigate and quantify the mechanical characteristics according to temperature and age [25]. Therefore, this study focused on preventing early frost damage using a newly developed insulated gang form instead of the method of improving concrete performance by adding a chemical admixture during concrete construction in winter, as in previous studies on protection in winter. In addition, the process of generating hydrates according to temperature and age was examined using a scanning electron microscope (SEM), and the micropore characteristics of concrete were investigated by mercury intrusion porosimetry (MIP).

## 2. Materials and Methods

### 2.1. Design of Experiments

**Figure 1:**
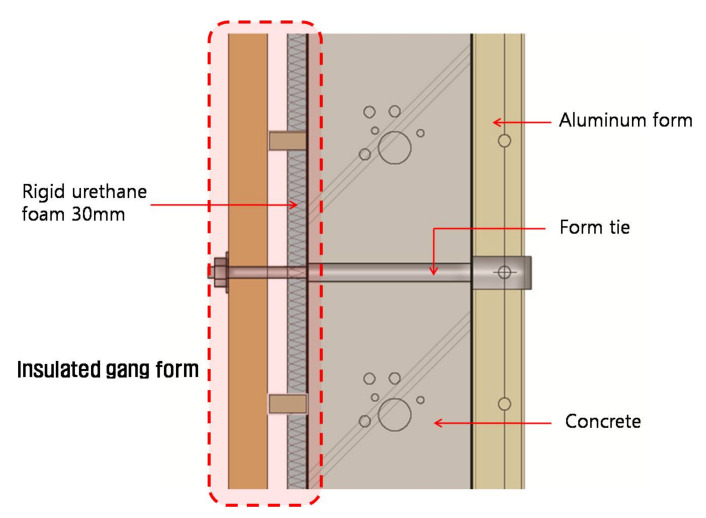
Schematic diagram of the insulated gang form (cross-section).

**Figure 2:**
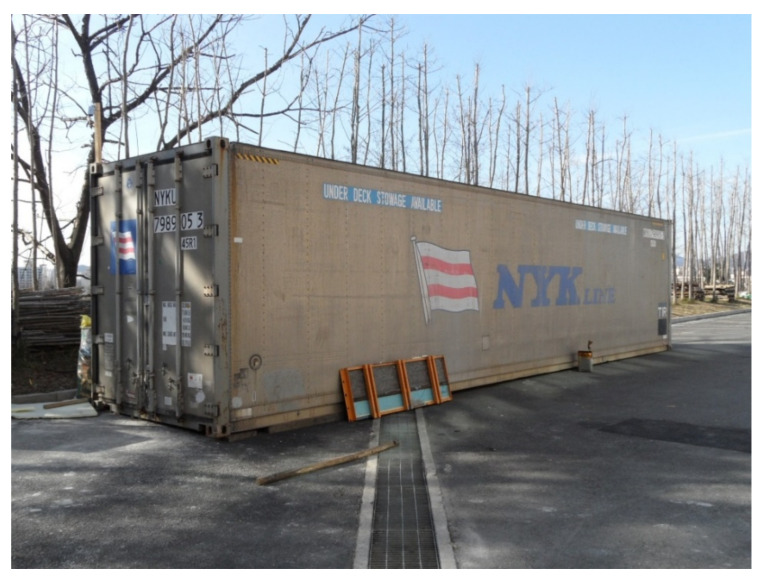
View of the refrigerated container.

The experimental design of this study is outlined in Table 1, and the detailed structure of the insulated gang form is illustrated in Figure 1. The experiment was performed under cold weather conditions with a minimum temperature of −20 °C. The insulated gang form used in the experiment consisted of a rigid urethane board (Development Advance solution Co.Ltd., Jeollanam-do, Korea) (insulation 30 mm) attached to the outside of the form with adhesives (Buildex Co.Ltd., Seoul, Korea.) The other variables and conditions are identical to those of the conventional gang form. Concrete was poured into this gang form, and it was compared with concrete fabricated following the conventional gang form to identify the temperature history, microstructure, and micropore characteristics. For accurate control of the outside air temperature in winter, the experiment was conducted in a refrigerated container, as shown in Figure 2.

The insulated gang form is prepared as a 100% factory-made product, and there is no need for additional on-site fabrication process such as spraying or affixing insulation Therefore, it is easy to install, with small human resources loss, and exhibits a higher insulation effect than the existing protection methods in winter.

For concrete, the typical concrete mix commonly used at construction sites in winter was used, without specifically adding admixture for facilitation of hydration reaction.

As for the water/binder ratio, the general strength combination, which is commonly used in construction sites, was used, and the target slump and air volume were set at 180 mm and 4.5 ± 1.5%. As for experimental variables, after concrete pouring, the slump and air content were measures. For hardened concrete, the temperature history, compressive strength, microstructure (SEM), and micropores of the concrete were measured.

The curing plan of this study considered an outside temperature in the range from 5 to −20 °C. The temperature inside the member was set to 5 °C or higher for three days after concrete pouring. Subsequently, the formwork was removed, and air curing was performed. The measured outside temperature ranged from −5 °C to 0 °C, which corresponded to the average temperature at the end of December (as in Asia–South Korea). For the conventional and insulted gang forms, a 3.0 mm-thick steel plate was used in addition to 50 mm × 30 mm × 2.3 mm rectangular pipes as vertical and horizontal members. A 30 mm-thick rigid urethane board was used as the insulation of the insulated gang form. Table 2 shows the concrete mix ratio, and Table 3 lists its physical properties.

### 2.2. Experimental Method

#### 2.2.1. Fabrication of Members and Measurement Method

**Figure 3:**
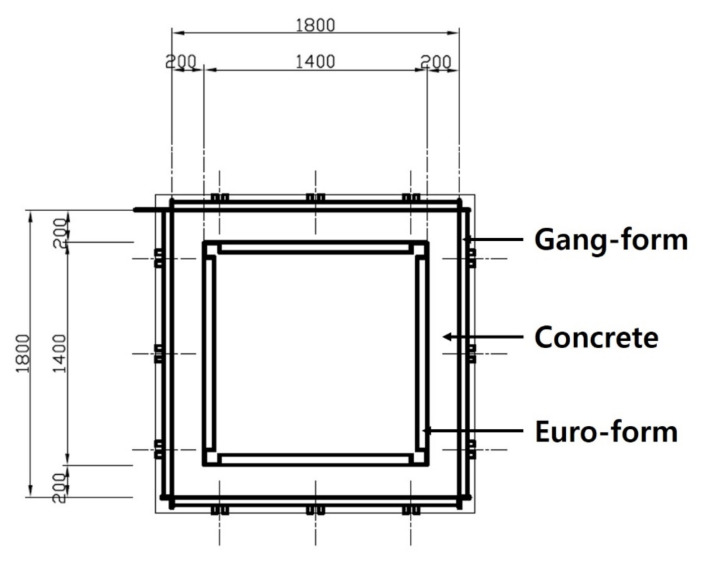
Sketch of the floor plan.

**Figure 4:**
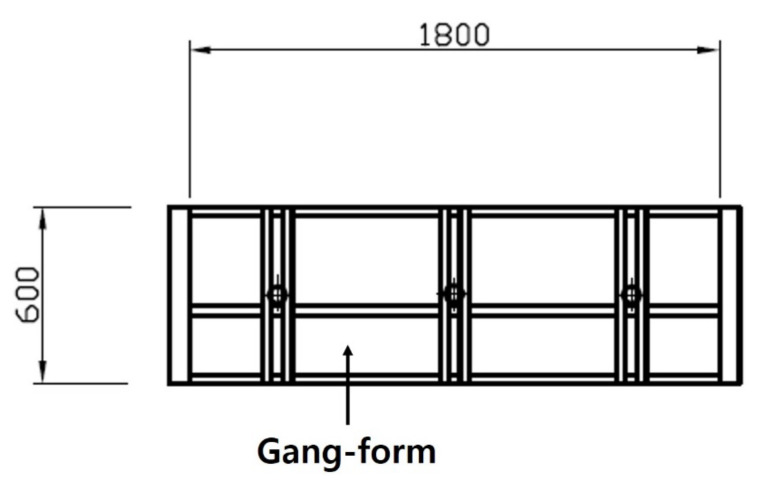
Sketch of the elevation.

For concrete, a ready-mixed concrete product, mainly used in general construction sites, was used. The experimental specimens were fabricated as a four-side closed box type with a wall thickness, width, and height of 200 m, 1800 mm, 600 mm, respectively, as shown in Figure 3 and Figure 4. The specimens were small-scale members in the most similar configuration to the insulated gang form actually constructed. In order to measure the temperature of the concrete, a T-type thermocouple (Omega Engineering Co.Ltd., Seoul, Korea) was fixed to the center and surface of each wall at a point of 300 mm in height, and an automatic temperature recorder (Omega Engineering Co.Ltd., Seoul, Korea) was set to record the results every 2 h. Temperature measurement was carried out immediately after placing the concrete.

**Figure 5:**
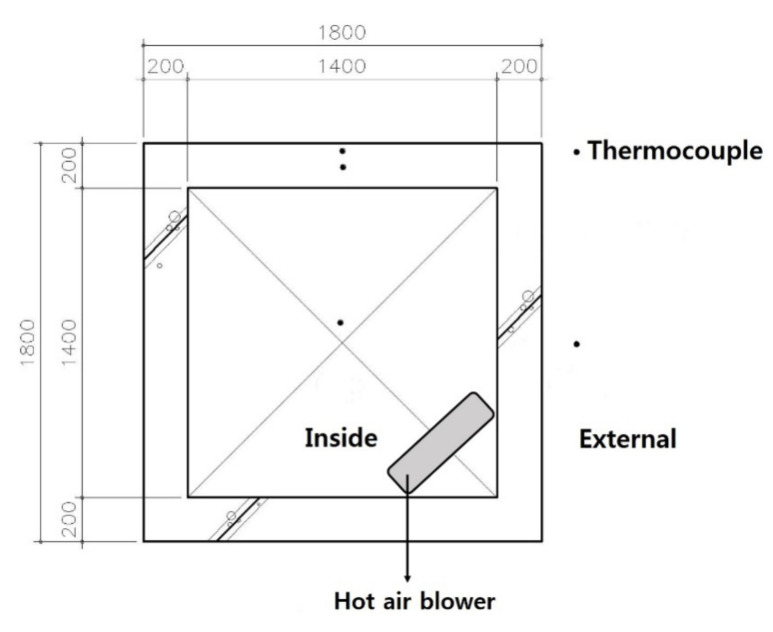
Thermocouple installation locations and placement of the hot-air blower.

The specimens were fabricated as a four-sided closed box type to have conditions similar to those at the actual construction site. Moreover, a hot-air blower (Osung Air Tech. Co.Ltd., Incheon-si, Korea) was installed, and the internal space was heated so that the internal temperature could be maintained at 5 °C. Figure 5 shows the thermocouple installation locations and placement of the hot-air blower.

Regarding the outside air condition for the specimens, curing was performed for three days by setting the temperature between 5 to −20 °C, which corresponds to cold weather conditions. Subsequently, the specimens were cured for 25 days at temperatures between −5 and 0 °C. This outside air condition was set based on the winter season in South Korea when insulated gang form is installed. The normal temperature condition (20 °C) was excluded from the experimental conditions as it did not meet the purpose of this study to prevent early freezing in winter.

In the preparation of specimen for SEM analysis and pore diameter measurement, the central part of the specimen was crushed at each day of age, the crushed parts were passed through a 5mm sieve, and small pieces remaining on the 2.5 mm sieve were extracted. In the concrete specimen, the core was taken from the central part of the structure to avoid other effects such as bleeding and settlement of aggregates. Acetone was used to stop the hydration reaction immediately after crushing the concrete core. As a method for stopping the hydration reaction, the specimen was immersed in acetone immediately, and the free water in the specimen was replaced with acetone. Acetone immersion was conducted for about 24 h, and new acetone was replaced every 8 h. When this process was completed, it was dried at 100 °C for 1 day using a dryer.

#### 2.2.2. Measurement with Scanning Electron Microscope (SEM)

**Figure 6:**
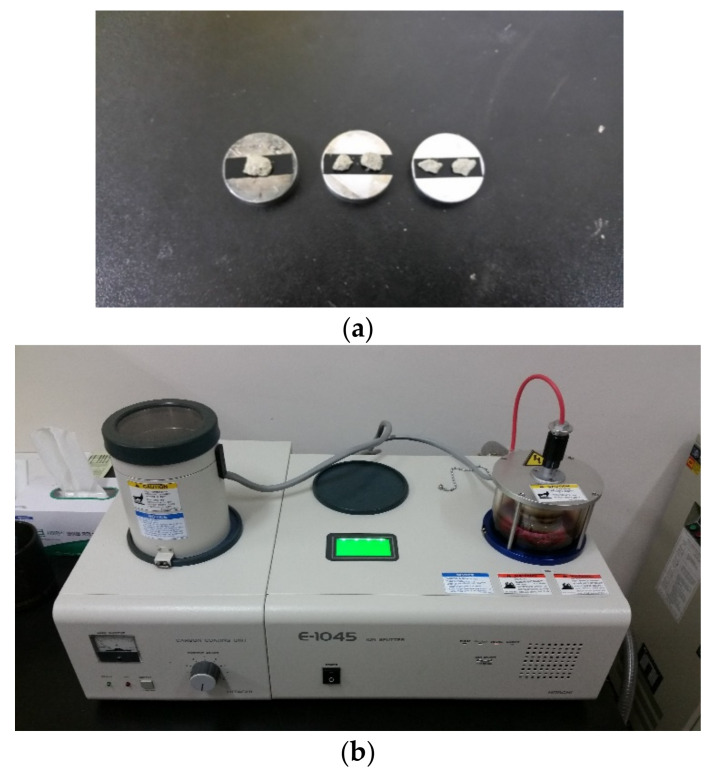
(**a**) Samples taken from the specimens. (**b**) Specimen surface treatment system (SEM).

The resolution of the SEM (Seron tech. Co.Ltd., Gyeonggi-do, Korea) is determined by how small the working distance of the electron beam and whether the beam can be applied to the specimen with high brightness. For use of SEM, a conductive material is coated on the surface of the specimen. In addition, for the surface of the specimen, a free fracture surface is desirable for a concrete structure or a hydration structure, and the surface should be appropriately flat. The specimen fixed with an adhesive or double-sided tape on the observation stand, and gold and carbon are deposited on the surface with a sputtering device such as an ion coater. The SEM observation is conducted in a vacuum of 0.075006 × 10^−^^3^ to 0.0075006 × 10^−^^3^ N/m^2^ with the coated specimen, and care is taken during the process to minimize the evaporation of the volatile component in the adhesive or double-sided tape used. In addition, cement pastes in the initial stage of hydration or colloidal substances need to be handled carefully. In this experiment, samples of about 5 mm were taken from the specimen as shown in Figure 6a and after surface treatment as shown in Figure 6b, the surface of the samples was observed at 10,000× magnification.

#### 2.2.3. Measurement of Pore Diameter Distribution by Mercury Intrusion

**Figure 7:**
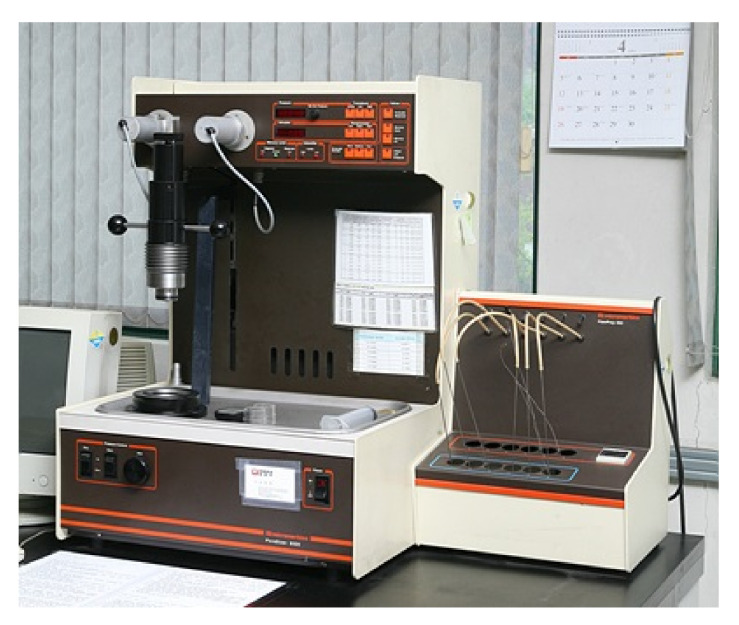
MIP distribution measuring device.

Concrete pore diameter distribution is affected by the materials used, mixing ratio, and curing method, but the total pore volume or pore diameter distribution of concrete varies depending on chemical degradation factors such as neutralization, sulfate corrosion, and acid rain or physical degradation factors such as frost damage. Due to such degradation, the durability of the concrete is reduced, but the measurements of pore diameter distribution are generally used for evaluation of various factors affecting the durability performance of the concrete rather than for quantitative diagnosis and evaluation of the concrete durability. That is, it is used as an indicator for evaluating various properties of concrete materials in many cases. As for the measuring tool in this experiment, pore distribution was measured using mercury intrusion with a porosimeter in Figure 7. The measurement range was 0.2 MPa, and the size range was 60A to 360 μm/Hg. In order to analyze the internal pore structure, calcium was eluted for 7 to 28 days of immersion age, and then micropore-size-distribution was analyzed by Mercury Intruction Porosimetry (MIP) (Micromeritics Co.Ltd., Atlanta, USA). As for the MIP analysis, three specimens were measured according to the elution time, and their uniformity was maintained by keeping the sample size to below 10 mm.

## 3. Results

### 3.1. Temperature History and Compressive Strength Characteristics

**Figure 8:**
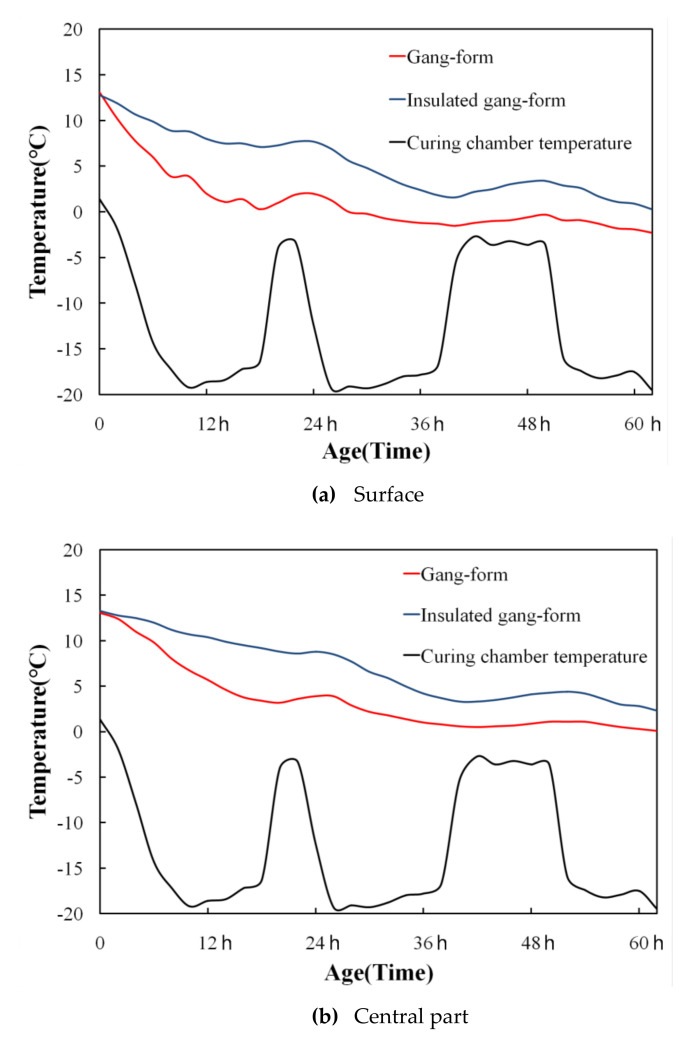
Temperature history of concrete.

Figure 8 shows the temperature history of the concrete for the gang form type on the external surface and in the central part. Immediately after concrete pouring, the heat of hydration showed a tendency to decrease for both the conventional and insulated gang forms because of the low outside temperature. However, the conventional gang form exhibited a faster decrease than the insulated gang form, and the concrete surface temperature dropped below 0 °C at 28 h of age.

The ideal temperature for concrete curing is 15 to 16 °C. The normal range of temperature for the concrete environment is between 5 and 32 °C [12]. At temperatures below 5 °C, the chemical reaction between cement and water is greatly reduced [12].

In the case of the insulated gang form, no significant increase was found in the heat of hydration due to the low outside temperature. However, the temperature history was higher than that of the conventional gang form. In addition, the temperature difference between the central and external surfaces was not large.

**Figure 9:**
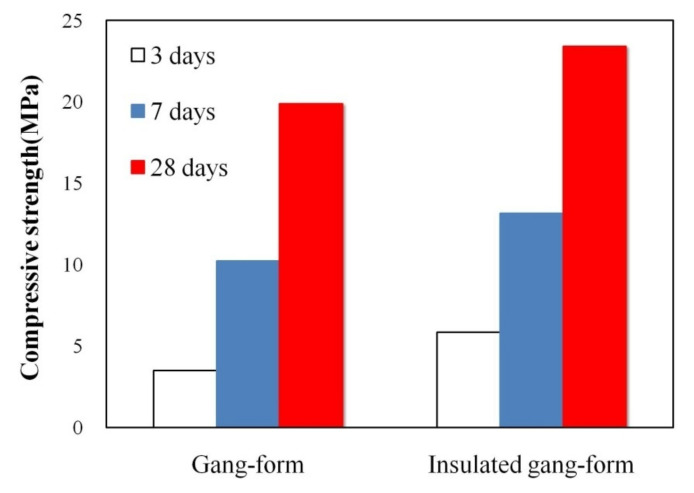
Compressive strength characteristics of concrete.

Figure 9 shows the compressive strength characteristics of concrete using the gang form type. The compressive strength of the conventional gang at 3 days of age was 3.51 MPa, which was lower than the compressive strength required for demolding (5 MPa). Nevertheless, in the case of the insulated gang form, the compressive strength at 3 days of age was 5.84 MPa, which exceeded the formwork stripping time and the value required to avoid early frost damage. Regarding the temperature history, the insulation performance of the insulated gang form was found to be excellent for improving the early compressive strength.

### 3.2. Microstructure Characteristics

The changes during the initial hydration reaction of cement are particularly pronounced within 28 days after hydration. In this process, calcium silicate hydrate (C-S-H) is the major products in addition to cement particles, and ettringite and monosulfate hydrates are also produced. In addition, relatively large calcium hydroxide crystals in the shape of a hexagonal plate can be seen at the early stage, which gradually decreases due to carbonation as well as the progress of hydration.

**Figure 10:**
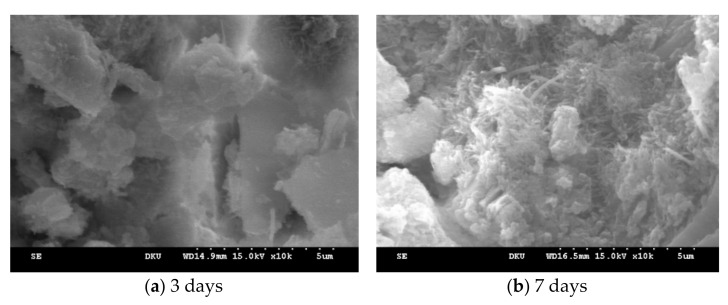
Microstructure characteristics (conventional gang form).

**Figure 11:**
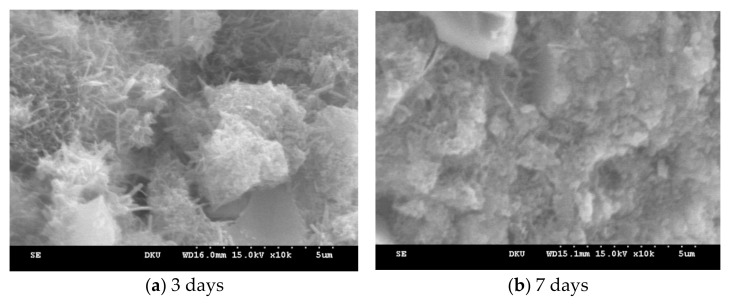
Microstructure characteristics (insulated gang form).

Figure 10 and Figure 11 show the microstructure analysis results by gang form type obtained by magnifying hardened cement 10,000 times through SEM. Figure 10a shows hydrated concrete in conventional gang form at 3 days of age. Since no hydration reaction occurred due to the low temperature of −20 °C, un-hydrated cement is mostly observed. However, it can be seen that from the 7 days of age in Figure 10b, ettringite in the form of needles is produced and slow process of hydration reaction can be observed.

At low temperatures, the hydration reaction starts very slowly, and ions dissolved before the precipitation of hydrates take longer time in diffusion and C-S-H hydrates with low density show more even distribution and small and irregular structure develop [26,27,28]. The solubility of ettringite increases significantly with temperature [27].

The interconnected needle shapes in Figure 11 a form the basis of the initial strength of the concrete, and more dense structure is formed over time. These needle shapes can only be observed at the initial stage of hydration, and these decrease over time as shown in Figure 11b.

However, when using the insulated gang form, the ettringite, C-S-H gel, and calcium hydroxide were generated at 3 days of age, indicating that its hydration reaction was better than that of the conventional gang form. At 7 days of age, the hydration products were denser than the conventional gang form.

Mostly ettringite is the initial hydration product, which is bridging the binder grains, until the stiffening and setting occurs [29].

The application of the insulated gang formed an accelerated hydration reaction owing to its excellent insulation performance; thus, it increased early compressive strength in addition to the temperature history and compressive strength characteristics shown in Section 3.1.

### 3.3. Micropore Characteristics

Irregular air voids in concrete cause reduced strength and concrete performance degradation. The fine pores in the hydration structure become even smaller as the hydration reaction proceeds, resulting in a denser microstructure.

**Figure 12:**
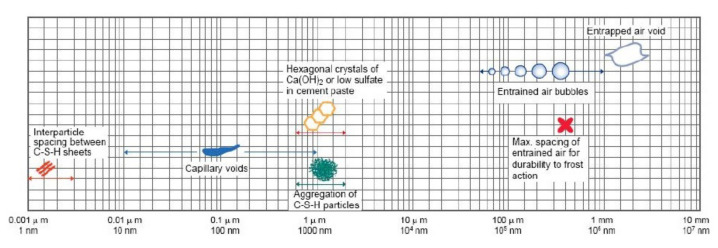
Dimensional range of solids and pores in a hydrated cement paste [30].

**Figure 13:**
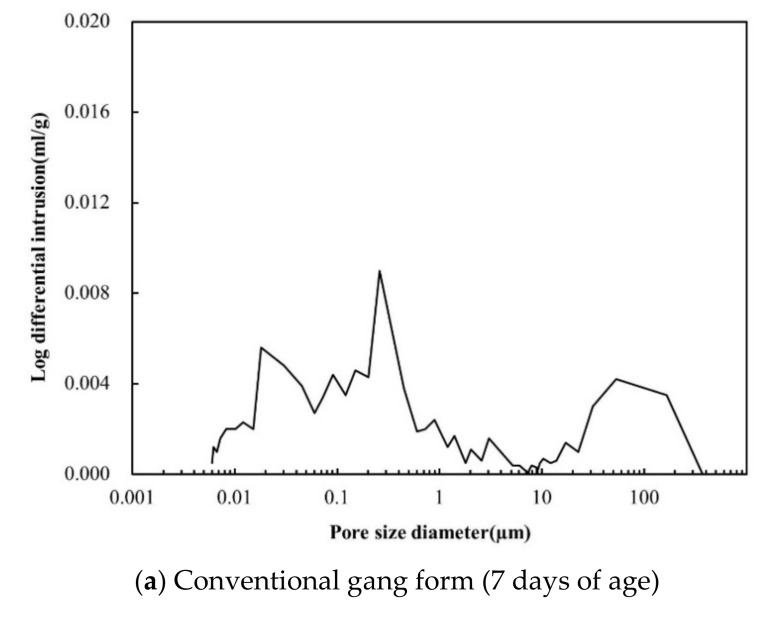

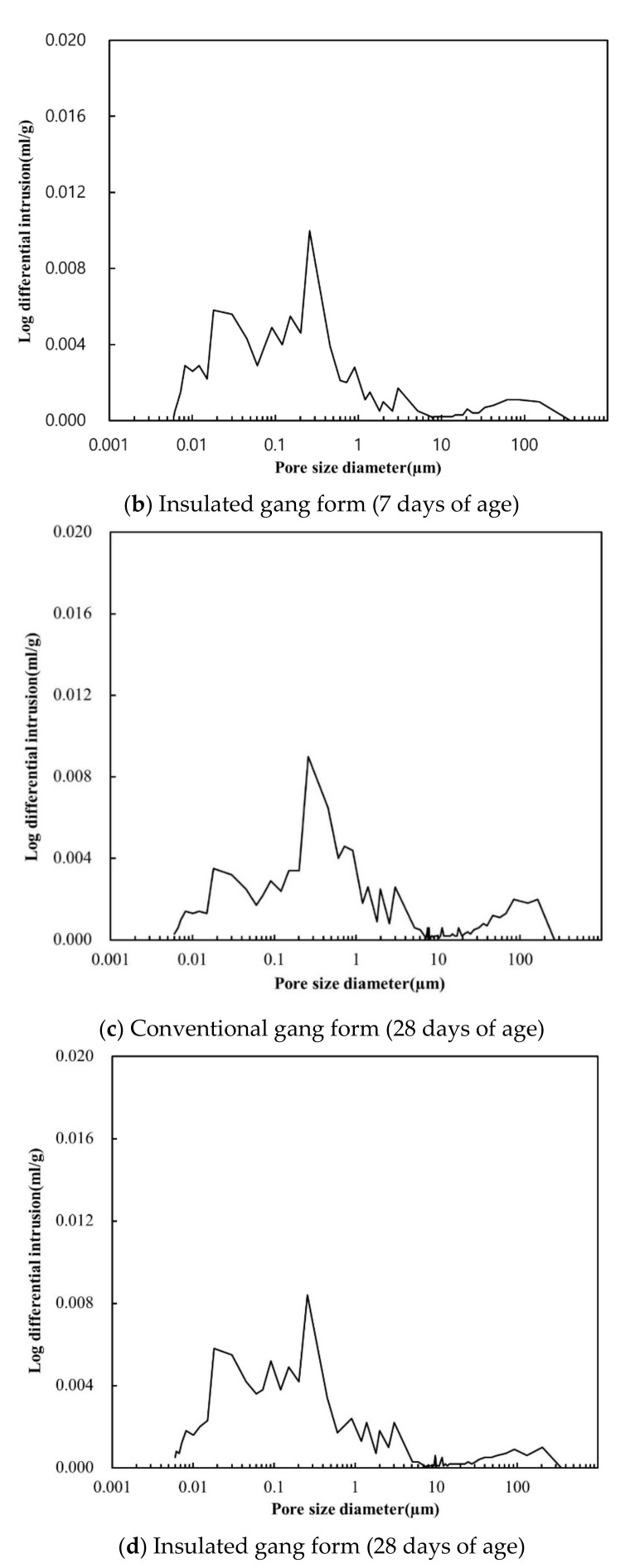
Pore structure at 7, and 28 days of age.

**Figure 14:**
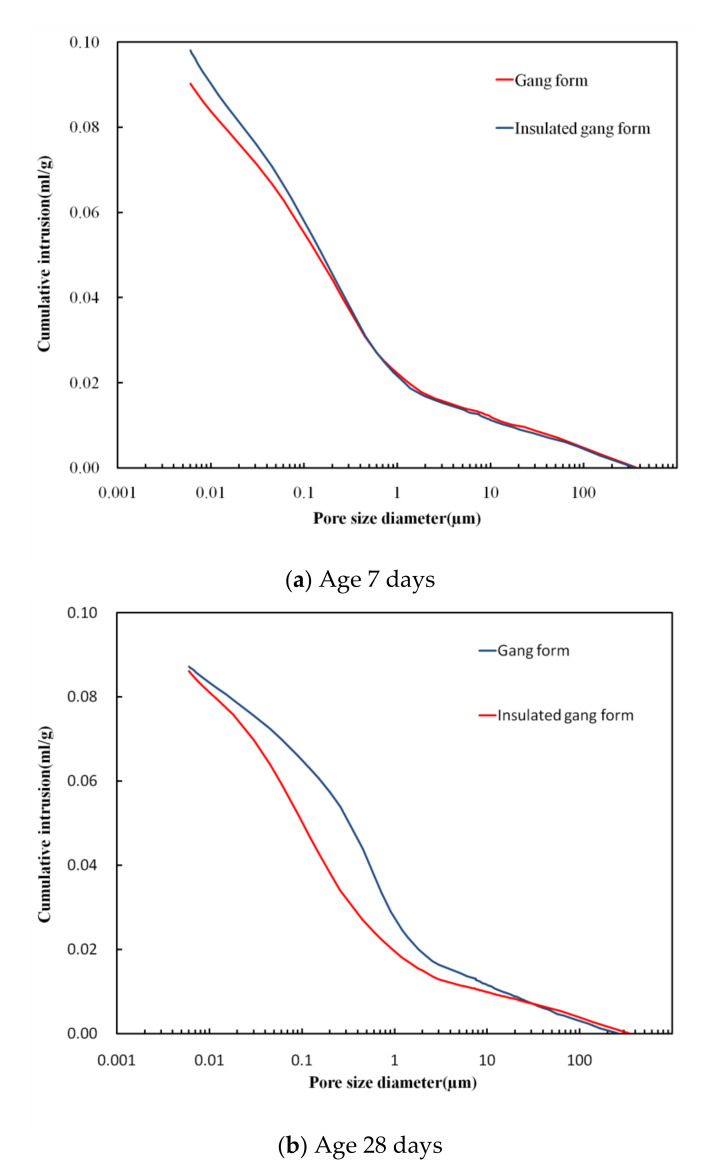
Cumulative pore volume.

Figure 12 shows the dimensional range of pores [30], and Figure 13 show the pore distribution inside the hardened cement, according to the gang form type after 7 and 28 days of age, obtained by MIP. Figure 14 shows the cumulative pore volume as function of the gang form type at 7 and 28 days of age. With an increase in the age, large pores of 100 µm in size decreases, and small pores of 1 µm or less increases in both conventional and insulated gang forms. This tendency was clear in the insulated gang form and in the conventional gang form.

As for the conventional gang form, the hydration reaction was slow due to low concrete retention, which resulted in larger unhydrated cement particles in the early age and irregular cement structure. Therefore, for the conventional gang form, relatively large pores between 50 and 300 µm were observed at 7 days of age owing to the low curing temperature. At 28 days of age, the distribution of large pores decreased because of the hydration reaction. However, in the insulated gang form, a smaller number of large pores of 100 µm were observed at 7 days of age than in the conventional gang form. A similar tendency was confirmed in the 0.01–1 µm range. Hydration reaction was faster in the insulated gang form than in conventional gang form at low temperatures with no special hydration accelerator added.

This is because, due to the development of hydration and pozzolanic reactions, precipitation of the primary and secondary hydration products occurred, filling the pores, and shifting the curve of pore size to a smaller size [31]. Prevention of early freezing is very important in curing concrete in winter. As suggested by the results of the experiment, the use of insulated gang form is expected to have a great effect in preventing early freezing damage by promoting hydration reaction and reducing pores (or water) compared to the conventional gang form in the early age. This result can be clearly seen in Figure 14b, which shows the 28-day total pore volume distribution curve, and the tendency is more clearly shown than the result in Figure 14a.

At 28 days of age, more small pores in the 0.01–0.1 µm range were observed in the insulated gang form than in the conventional gang form, indicating superior micropores characteristics than when using the conventional gang form. In particular, pores smaller than 0.05 µm are known to be closely related to concrete strength because they are in the sizes of gel pores and capillary pores, as shown in Figure 13. The application of the insulated gang form slightly increases micropores with sizes close to 0.05 µm, compared to the conventional gang form. These results indicate that the insulated gang form improves concrete strength in cold weather [30]. As the hydration reaction proceeds, the fine pores inside the hydration tissue becomes smaller and form a more dense structure. This phenomenon has a great effect on improving strength and preventing deterioration.

The pore size distribution has a great impact on the durability of concrete. From the size distribution, large pores have a negative effect on the durability of the concrete. However, when pores are uniformly introduced as in the use of air-entraining (AE) agent, the concrete resistance to freezing and thawing improves.

The results showed that the sample that used the insulated gang form exhibited a smaller cumulative pore volume than the samples that used the conventional gang form as the age increased.

This experimental result shows the comparative analysis on the cement hydration results according to the insulation performance of the insulated gang form. The analysis according to the secondary cement-product, admixture, and aggregate type is not considered in this result. Further studies need to be conducted reflecting these details in the future.

## 4. Conclusions

The experiment in this study was performed under cold weather conditions with a minimum temperature of −20 °C. When the insulated gang form was applied under such conditions, the temperature history, compressive strength, microstructure, and micropore characteristics of concrete were analyzed. The main results are as follows:

1. Immediately after concrete pouring, the conventional gang form exhibited a faster decrease in the heat of hydration than the insulated gang form, and the concrete surface temperature dropped below 0 °C at 28 h of age. These results are likely to have a great influence on the initial curing of concrete.
2. The compressive strength of the conventional gang form did not exceed 5 MPa at 3 days of age. However, for the insulated gang form, the compressive strength at 3 days of age exceeded 5 MPa. This greatly contributes to the shortening of the construction period due to the rapid demolding time of the formwork.
3. When the microstructure was analyzed according to the member type, a large amount of unhydrated cement was observed in the conventional gang form at 3 days of age because the hydration reaction was barely performed because of the low temperature. However, in the insulated gang form, ettringite, C-S-H gel, and calcium hydroxide, which are hydration products, were observed in large quantities at 3 days of age. These results indicate that the hydration reaction was further accelerated compared to the case when the conventional gang form was used.
4. As age increased, large pores decreased and pores smaller than 1 µm increased. In addition, the distribution of relatively small pores increased in the insulated gang form compared to that in the conventional gang form. Overall, the insulated gang form exhibited a smaller cumulative pore volume than the conventional gang form as the age increased.

Summarizing the results of this study, the use of insulated gang form for the construction of apartment houses in cold winter will result in a denser concrete structure than that with the conventional gang form to ensure the concrete quality in winter.

## Figures and Tables

**Table 1:** Experimental design: variables, number, and level.

Experimental Variable	Experimental Number	Experimental Level
w/b	1	0.499
Slump (mm)	1	180
Air (%)	1	4.5 ± 1.5
Curing chamber minimum/maximum temperatures (°C)—3 days	2	−20/5
Temperature inside the member (°C)	1	5
Hardened concrete	4	Temperature history, compressive strength, microstructure (SEM), and micropore.

W. w/b: water/binder (cement + fly ash + blast furnace slag) ratio.

**Table 2:** Concrete mix ratio.

w/b	W (kg/m^3^)	S/a (%)	Unit Weight (kg/m^3^)					
			C	B	F	S	G	SP
0.499	171	48	275	34	34	870	951	2.4

W: unit water content, S/a: fine aggregate ratio, C: Portland cement. S: fine aggregate, G: coarse aggregate, B: blast furnace slag, F: Fly-ash, SP: Super Plasticizer.

**Table 3:** Physical properties of the urethane board used in insulated gang form.

Purpose	Species	Ingredient	Thickness (mm)	Thermal Conductivity (W/m·k)	Density (kg/m^3^)
Insulation	Polyurethane	Polyol + Isocyanate	30	0.018	35

## Data Availability

Not applicable.
